# Expansion of induced pluripotent stem cells under consideration of bioengineering aspects: part 2

**DOI:** 10.1007/s00253-024-13373-2

**Published:** 2025-02-06

**Authors:** Misha Alexander Teale, Samuel Lukas Schneider, Stefan Seidel, Jürgen Krasenbrink, Martin Poggel, Dieter Eibl, Marcos F. Q. Sousa, Regine Eibl

**Affiliations:** 1 Centre for Cell Cultivation Techniques, Tissue Engineering, and Medical Biology, Institute of Chemistry and Biotechnology, ZHAW Zurich University of Applied Sciences, Grüentalstrasse 14, 8820 Wädenswil, Switzerland; 2 Advanced Manufacturing-Platform Engineering and Support, Bayer AG, Kaiser-Wilhelm-Allee 1, 51373 Leverkusen, Germany

**Keywords:** Adherent cell culture, Allogeneic, Fixed-bed bioreactor, Multiplate bioreactor, Perfusion, Single-use bioreactor

## Abstract

The manufacturing of allogeneic cell therapeutics based on human-induced pluripotent stem cells (hiPSCs) holds considerable potential to revolutionize the accessibility and affordability of modern healthcare. However, achieving the cell yields necessary to ensure robust production hinges on identifying suitable and scalable single-use (SU) bioreactor systems. While specific stirred SU bioreactor types have demonstrated proficiency in supporting hiPSC expansion at L-scale, others, notably instrumented SU multiplate and fixed-bed bioreactors, remain relatively unexplored. By characterizing these bioreactors using both computational fluid dynamics and experimental bioengineering methods, operating ranges were identified for the Xpansion^®^ 10 and Ascent™ 1 m^2^ bioreactors in which satisfactory hiPSC expansion under serum-free conditions was achieved. These operating ranges were shown not only to effectively limit cell exposure to wall shear stress but also facilitated sufficient oxygen transfer and mixing. Through their application, almost 5 × 10^9^ viable cells could be produced within 5 days, achieving expansion factors of up to 35 without discernable impact on cell viability, identity, or differentiation potential.

**Table:** 

**Key Points** •*Bioengineering characterizations allowed the identification of operating ranges that supported satisfactory hiPSC expansion* •*Both the Xpansion*^®^ *10 multiplate and Ascent™ 1 m*^2^ *fixed-bed reactor accommodated the production of almost 5 × 10*^9^ *viable cells within 5 days* •*Exposing the hiPSCs to a median wall shear stress of up to 8.2 × 10*^−5^ *N cm*^−2^ *did not impair quality*

**Supplementary Information:**

The online version contains supplementary material available at 10.1007/s00253-024-13373-2.

## Introduction

The manufacturing of allogeneic cell therapies (CT’s) based on human induced pluripotent stem cells (hiPSCs) presents a significant step towards facilitating accessible and affordable healthcare for a wide range of clinical indications (Weed and Mills 2017; Gunhanlar et al. 2018; Laco et al. 2020; Hogrebe et al. 2021). However, given the demands placed on manufacturing, with between 10^5^ and 10^12^ cells required per dose to ensure adequate treatment (Scibona and Morbidelli 2019), SU bioreactors capable of achieving such yields remain to be conclusively identified and characterized.

Commercially available L-scale SU bioreactors, which have already proven themselves suitable for the expansion of adherent stem cells, include both conventional non-instrumented static cultureware (Tohyama et al. 2017) and instrumented dynamic bioreactors. Given the importance of instrumentation (Pandey et al. 2020; Manstein et al. 2021), reproducibility (Schirmaier et al. 2014; Huang et al. 2020), scalability (Cameau et al. 2019; Gautam et al. 2023), and yield (Scibona and Morbidelli 2019) for the production of allogeneic hiPSC-based CTs, dynamic single-use (SU) bioreactors, mixed either mechanically (Sousa et al. 2015; Jossen et al. 2016; Kwok et al. 2018; Pandey et al. 2020; Huang et al. 2020; Cohen et al. 2023; Schneider et al. 2025) or hydraulically (Lambrechts et al. 2016b, 2016a; Haack-Sørensen et al. 2018; Paccola Mesquita et al. 2019; Mennan et al. 2019; Vymetalova et al. 2020; Rasby and Barker 2022), are to be preferred over their static counterparts. Among the mechanically driven SU bioreactors, stirred bioreactors are especially well-studied from a bioengineering perspective (Schirmaier et al. 2014; Lawson et al. 2017; Borys et al. 2021; Schneider et al. 2025)  and have already demonstrated the ability to produce up to 20 × 10^9^ hiPSCs within 15 days when cultivating the hiPSCs on microcarriers or as spheroids (Pandey et al. 2020; Huang et al. 2020).

On the contrary, little has been reported on hiPSC expansion in hydraulically driven SU hollow-fiber (Paccola Mesquita et al. 2019), multiplate, and fixed-bed bioreactors, especially in connection with relevant bioengineering parameters, such as wall shear stress ($\tau$). This is particularly noteworthy, given that the exposure of adherent cells to $\tau$ ≥ 100 × 10^−5^ N cm^−2^ has been shown to promote cell detachment in the absence of a proteolytic reagent (Fuhrmann and Engler 2015), risking potential hiPSC loss through dissociation-induced apoptosis (Watanabe et al. 2007). Moreover, given the importance of cell quality (Sullivan et al. 2018), a $\tau$ ≥ 10 × 10^−5^ N cm^−2^ may already be considered sufficiently detrimental to the cultivation of hiPSCs, as it has been shown to influence pluripotent stem cell identity (Huang et al. 2021). Alongside $\tau$, other bioengineering parameters, such as mixing time (${\theta }_{M}$) and the volumetric mass transfer coefficient (${k}_{L}a$), must also be considered, as both influence localized oxygen gradients, and, therefore, directly impact stem cell yield and quality, irrespective of bioreactor used (Sousa et al. 2015; Dashtban et al. 2021). As such, while hypoxic conditions are favorable when cultivating stem cells (Mas-Bargues et al. 2019; Nit et al. 2021), prolonged exposure to severe hypoxia or even anoxia results in genetic instability and apoptosis (Riffle and Hegde 2017; Deynoux et al. 2020; Nit et al. 2021).

**Fig. 1:**
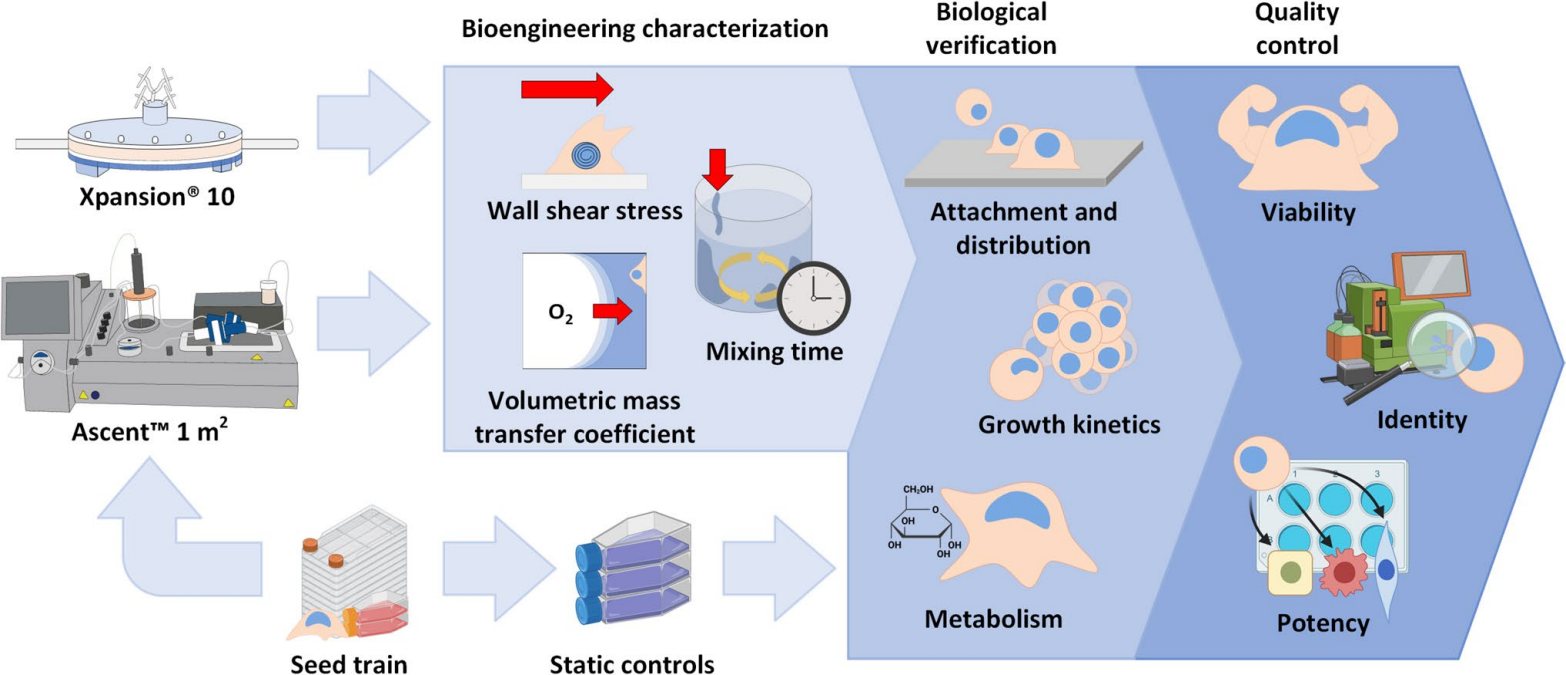
Experimental workflow for successfully expanding hiPSCs in the XP10 and fixed-bed AS1 bioreactors under serum-free conditions. Both L-scale SU bioreactors were characterized using numerical and experimental bioengineering methodologies, allowing suitable operating ranges to be identified for the bioengineering parameters within which hiPSC growth and quality would be maintained. The suitability of the operating ranges was subsequently confirmed through biological experiments where > 10^9^ hiPSCs were produced under serum-free conditions without loss of quality. Alongside the dynamic cultivation in the XP10 and AS1, static T-flask cultures served as positive controls for cell growth and quality. All biological experiments were monitored through daily sampling, while hiPSC quality was determined and compared to the T-flask controls by assessing viability, identity, and potency directly prior to inoculation and following harvest. Image partially created with Biorender.com

This article describes, for the first time, the characterization of the SU Xpansion^®^ multiplate and Ascent™ fixed-bed bioreactors using both numerical and experimental methodologies, thereby allowing suitable operating ranges to be identified for the bioengineering parameters within which hiPSC growth and quality would be maintained (Fig. 1). The suitability of the defined operating ranges was then confirmed through biological experiments and the production of > 10^9^ hiPSCs under serum-free conditions without loss of viability, identity, and differentiation potential.

## Materials and methods

### Bioengineering characterization of the bioreactors

**Fig. 2:**
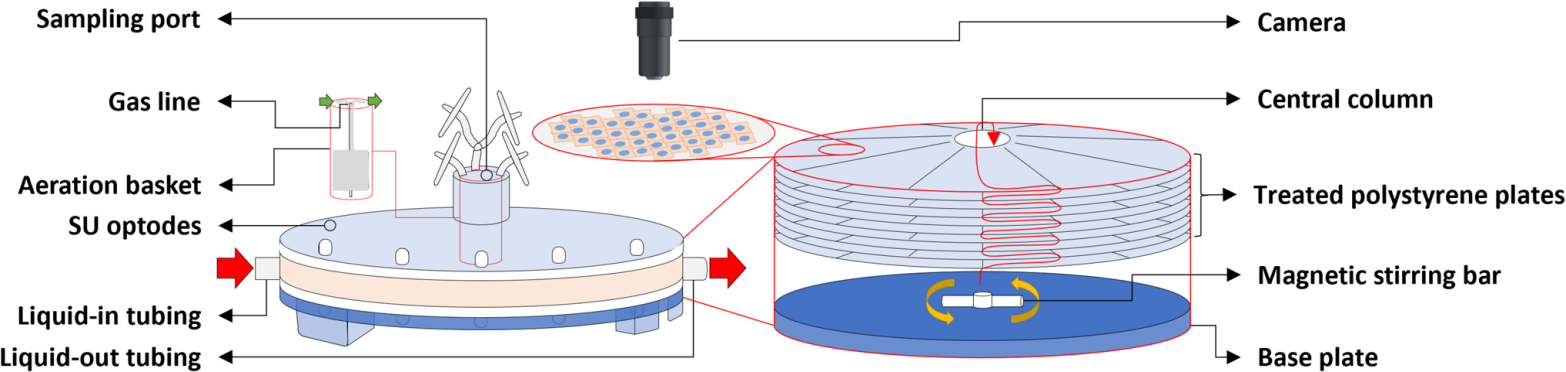
A simplified illustration of the XP10 SU bioreactor. The XP10 offers ≈ 6120 cm^2^ of cultivation surface area, comprising ten tissue culture (TC)–treated polystyrene plates. These plates are arranged around a central column within which the aeration basket is housed. While the plates serve as a scaffold for cell attachment and proliferation, the silicone membrane of the aeration basket acts as an interface for bubble-free gas exchange. Liquid addition and removal are facilitated through tubing located on either side of the bioreactor’s base plate, while SU optodes on the head plate allow for inline monitoring and regulation of pH and dissolved oxygen (DO). Therefore, for the bioreactor to function as intended, all plates must be fully submerged, resulting in a narrow working volume (${V}_{L}$) range of 1.5–1.6 L. When active, the magnetic stirring bar pushes the conditioned medium along the channels of the base plate and up through the radial channels of the treated plates, thereby supplying the resident adherent cells with O_2_ and nutrient-rich medium. Once the topmost plate has been reached, the spent medium passes the integrated optodes and recirculates back towards the stirrer via the central column. The bioreactor accommodates perfusion mode operation by continuously exchanging medium using the liquid-in and -out lines

**Fig. 3:**
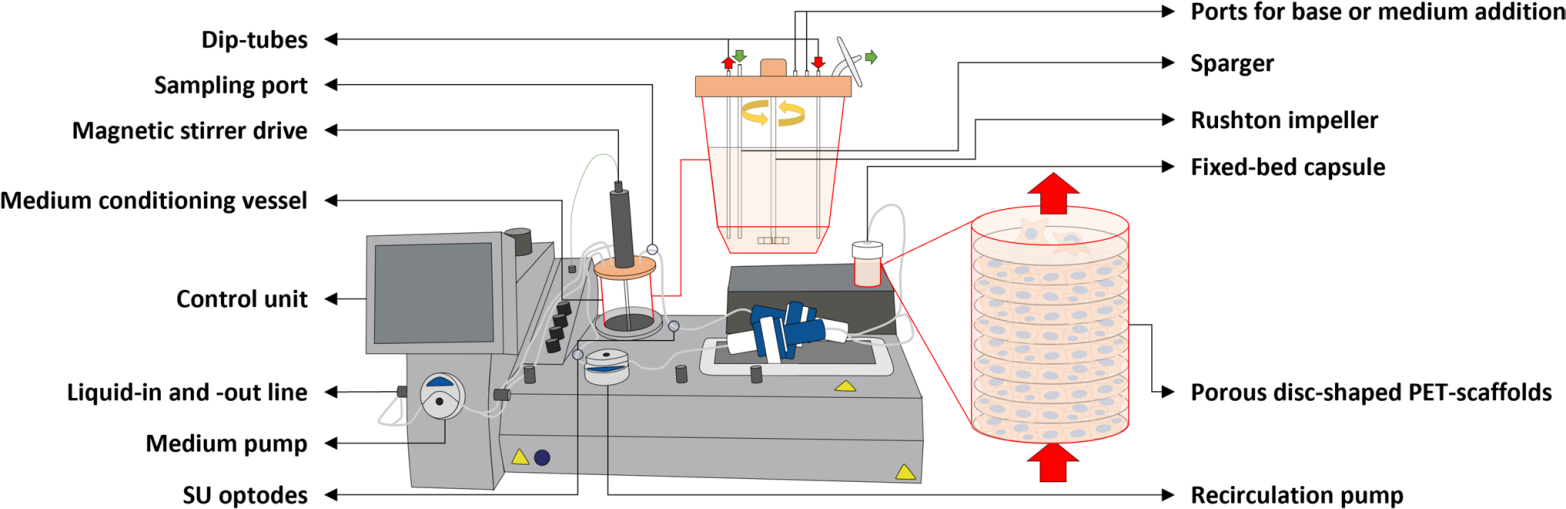
A simplified illustration of the AS1 SU bioreactor. The bioreactor consists of two SU compartments, each serving a distinct function. During operation, the medium conditioning vessel (MCV) with a ${V}_{L}$ of up to 3.5 L conditions the medium while cells adhere to and grow on the FBR’s ≈ 145 woven disc-shaped polyethylene terephthalate (PET)-scaffolds (total growth surface ≈ 1 m^2^). Adequate mixing is ensured in the MCV through a six-bladed Rushton impeller, with DO and pH regulated by gassing through a single integrated open-pipe sparger. Continuous bi-directional bubble-free O_2_ and nutrient-rich medium recirculation between the compartments is realized through a single peristaltic pump and two dip tubes. The medium exchange within the MCV is further accommodated through a second pump and corresponding liquid-in or -out line. SU optodes and sensors located along the recirculation loop permit inline monitoring and regulation of DO, pH, and temperature, while ports on the head plate of the MCV allow for base and medium addition

As described in greater detail by Schneider et al. 2025, both the Xpansion® 10 multiplate (XP10) [Fig. 2] (Cytiva, US) and Ascent™ 1 m^2^ fixed-bed (AS1) [Fig. 3] (Corning Inc., US) bioreactors were hydrodynamically characterized using computational fluid dynamic (CFD) simulations. System geometry was mapped using 3D scanning (EinScan Pro, Shining 3D Tech. Co. Ltd., CN), classical measurement, and the software Autodesk Inventor Professional 2023 (Autodesk Inc., US). All simulations were conducted based on the Navier–Stokes equations. Given that both bioreactor compartments contining the cells are completely filled with liquid and are operated bubble-free, single-phase simulations were carried out. For both systems, it was assumed that a quasi-stationary fluid flow profile would occur; therefore, the simulations were carried out as steady-state simulations (Werner et al. 2014). Furthermore, as the modified Reynolds number for the evaluated process parameters did not suggest the presence of turbulent flow, the simulations were performed under the assumption of laminar flow, without the inclusion of a turbulence model.

The rotation of the XP10’s stirrer was modeled using the Multiple Reference Frame method, as previously described in Schneider et al. (2025). Given the complexity of the AS1’s fixed-bed reactor (FBR) capsule and scaffold, two modeling approaches consisting of a macro- and microscopic part were required. The macroscopic simulation modeled the entire FBR capsule, simplifying the complexity of the scaffold by treating it as a porous zone. The pressure drop in the porous zone was modeled using the Darcy-Forchheimer equation, whereby the coefficients were determined experimentally using pressure and flow sensors over a specific scaffold length. For the microscopic simulation, $\tau$ within the FBR’s scaffold was spatially resolved by accounting for the liquids velocity (${U}_{L}$) within the geometry of the scaffold.

All simulations assumed a no-slip boundary condition for all bioreactor walls, stirrers, and internals. Furthermore, all simulations were performed at 37 °C, which corresponds to the temperature of the liquid phase during the experimental operation. At this temperature, the liquid phase was assumed to have a density ($\rho$) and kinematic viscosity ($v$) of 993.4 kg m^−3^ and 0.696 × 10^−6^ m^2^ s^−1^, respectively. All CFD simulations were performed on a high‐performance computing system using OpenFOAM version 10 (OpenFOAM Software, UK) as described in Seidel and Eibl (2021), with Paraview 5.10 (Kitware Inc., US) and Python 3.10 (Python Software Foundation, US) used for post-processing purposes. Following simulation, $\tau$ was specifically calculated for the bioreactor surfaces where cell adhesion could occur. For the XP10, this was accomplished by filtering the surface normals. $\tau$ was calculated using Eq. (1), where $v$ represents the liquids kinematic viscosity, $\rho$ the density, $y$ the distance in the normal direction, and ${U}_{L}$ the fluid velocity.

1 $$\tau =v\times \rho \times {\left(\frac{\partial {U}_{L}}{\partial y}\right)}_{y=0}$$

The results of the numerical simulations were then compared with either reported (Yablonsky et al. 2021) or experimental values, with the latter produced under select process conditions according to accepted methodologies (Bauer et al. 2020). These methodologies further facilitated the characterization of ${\theta }_{M}$, ${k}_{L}a$, and residence time distribution (RTD) within the XP10 and AS1’s MCV. While a detailed description of how ${\theta }_{M}$ and ${k}_{L}a$ were determined may be found elsewhere (Bauer et al. 2020) Schneider et al. (2025), the comparably high ${\theta }_{M}$ reported for the XP10 (Yablonsky et al. 2021) prompted additional RTD experiments, as described by Neumann et al. (2014), to confirm the absence of channeling and dead zones when operating in perfusion mode. For these experiments, an aqueous KCl solution with a conductivity of ≈ 3000 µS cm^−1^ (${\kappa }_{\infty }$) was introduced at a dilution rate ($D$) of 0.7 day^−1^ via the XP10s liquid-in port at a temperature of 37 °C and a stirring speed ($N$) of 42 rpm. Conductivity at time ($t$) was then measured at the XP10’s liquid-out port (${\kappa }_{t}$), which, together with an initial conductivity (${\kappa }_{0}$) measurement of ≈ 1000 µS cm^−1^, allowed the calculation of non-dimensional time-dependent F-curve or $F(t)$ as shown in Eq. (2).

2 $$F\left(t\right)=\frac{{\kappa }_{t}-{\kappa }_{0}}{{\kappa }_{\infty }-{\kappa }_{0}}$$

A comparison between the ideal and experimentally determined RTD was then made by calculating both the mean residence time ($\overline{t }$) and its variance (${\sigma }^{2}$), using Eqs. (3) and (4), respectively.

3 $$\overline{t }={\int }_{0}^{\infty }(1-F(t))dt$$

4 $${\sigma }^{2}=2\times {\int }_{0}^{\infty }\left(1-F\left(t\right)\right)dt-{\overline{t} }^{2}$$

### Cell line and seed train preparation

As described by Schneider et al. (2025), the seed train (ST) was prepared using the commercially available Gibco™ Episomal TMOi001-A hiPSC line (Thermo Fisher Scientific Inc., US). The hiPSCs were plated at 1.0–2.5 × 10^4^ cells cm^−2^ on recombinant human vitronectin (rhVTN)-coated, TC-treated cultureware (Corning Inc., US) and subsequently expanded under serum-free conditions using either Essential 8™ Flex [E8F] (Thermo Fisher Scientific Inc., US) or mTesR1™ [MR1] (STEMCELL Technologies, US). The medium was supplemented for the first 24 h with Y-27632 [RI] (Miltenyi Biotec, DE), with regular medium exchanges performed to either remove RI or replenish essential nutrients and growth factors. Before reaching a confluence of > 85%, the hiPSCs were passaged either as clumps or as single cells using either Versene™ (Thermo Fisher Scientific Inc., US) or Accutase^®^ (Corning Inc., US or STEMCELL Technologies, US), respectively, as described by Lai et al. (2022). Following detachment, the harvest reagent was quenched, the cell suspension spun down, the resulting supernatant discarded, and the cells resuspended in RI-supplemented culture medium in preparation for subsequent inoculation. In this manner, the cells were passaged at least twice prior to single-cell inoculation of the various bioreactor systems.

### Bioreactor preparation and operation

#### The Xpansion 10 multiplate bioreactor

Coating of the XP10’s treated plates was accomplished by exposing the interior of the bioreactor to a 1.9 µg mL^−1^ (0.5 µg cm^−2^) buffered rhVTN solution for > 12 h. Parallel to the coating procedure and prior to inoculation, the XP10 and 1.6 L of RI-supplemented cultivation medium were equilibrated at 37 °C, 5% CO_2_. The spent coating solution was then drained from the XP10 and replaced within ≈ 5 min using a peristaltic pump, thus achieving a final ${V}_{L}$ of 1.6 L, a working volume-to-surface area ratio (${V}_{L}/A$) of ≈ 0.25 mL cm^−2^, and a viable cell density ($VCD$) of 7.5–8.0 × 10^4^ cells mL^−1^ (1.9–2.1 × 10^4^ cells cm^−2^). In contrast to what has been previously reported (Lambrechts et al. 2016b), the cell density of the inoculum was not corrected to account for void volume.

Once inoculated, the bioreactor was neither gassed nor stirred for 4 h to promote hiPSC attachment to the treated plates. Thereafter, pH and DO were regulated to a setpoint of 7.2 and 40%, respectively, by gassing CO_2_, N_2_, O_2_, and air at a combined flow rate (${F}_{c}$) of 60–140 mL min^−1^ through the XP10’s aeration basket while stirring at a $N$ of 42–80 rpm. As supported by the findings of Schneider et al. (2025) and suggested by the manufacturer (Pall 2020), the dilution of RI, alongside improved pH and DO control, was facilitated through the perfusion of fresh medium at a $D$ of 0.7–1.4 day^−1^ (185–370 μL cm^−2^ day^−1^) following a 24-h batch phase. During perfusion mode operation, a constant ${V}_{L}$ was maintained either by implementing a gravimetric control loop or by adding a SU check valve to the waste line and implementing a bleed-to-pressure approach.

After 4–5 days, single cell harvest was performed to quantify cell yield, viability, identity, and differentiation potential. Briefly, the spent medium was removed from the XP10 via the tubing at the base of the bioreactor. The bioreactor was then filled with harvest solution, prepared as previously described Schneider et al. (2025), and incubated for 20–25 min at 20–25 °C. Towards the end of the incubation period, cell detachment was further assisted through mechanical shaking of the XP10 using its harvest station. The detached hiPSCs were then pumped into a collection vessel, and the bioreactor flushed with cultivation medium to quench the harvest solution and improve cell recovery. Harvest efficiency ($HE$) or cell recovery (Narumi et al. 2020) was then determined by estimating theoretical cell yield based on the confluence-to-cell density relationship determined during the T-flask experiments (Schneider et al. 2025) and comparing it to the final yield.

#### The Ascent™ 1-m^2^ fixed-bed bioreactor

Given the differences between the chemical composition of the AS1’s and XP10’s scaffold, three potential cell adhesion mediators (CAMs), namely rhVTN, Synthemax II (SynII) (Corning Inc., US), and recombinant human laminin-521 (rhBL) (BioLamina, SE), were identified following a review of current literature (Badenes et al. 2016; Miyazaki et al. 2017; Sivalingam et al. 2021; Dias et al. 2022) and selected based on the results of preliminary screening experiments (Figure S1). These CAMs were then used to coat the AS1’s FBR capsule by continuously recirculating solutions containing either 0.5 µg cm^−2^ (1.9 µg mL^−1^) of rhVTN, 5 µg cm^−2^ (100 µg mL^−1^) of SynII, or 0.2 µg cm^−2^ (3.3–5 µg mL^−1^) of rhBL for > 6 h at 60 mL min^−1^ and 20–25 °C. During this time, 0.5–0.6 L of RI-supplemented cultivation medium was added to the MCV in preparation for medium conditioning and subsequent inoculation. Once coated, the FBR was flushed with either phosphate-buffered saline (PBS) or medium, after which the volumetric flow rate of the liquid ($Q$) between the MCV and FBR was set to 60 mL min^−1^, corresponding to the lowest flowrate recommended by the manufacturer. Medium conditioning to a DO of 40% and a pH of 7.2 was realized over > 4 h by sparging a mixture of CO_2_, N_2_, O_2_, and air directly into the MCV while operating at 37 °C.

A starting ${V}_{L}$ of 0.7–0.8 L was achieved by adding 0.1–0.3 L of RI-supplemented cultivation medium containing 0.9–2.4 × 10^6^ cells mL^−1^ (2.0–3.0 × 10^4^ cells cm^−2^) either directly to the MCV or via the FBR. During direct MCV inoculation, the recirculation pump was stopped for ≈ 5 min to allow for the homogenous distribution of cells within the MCV at a $N$ of 60 rpm before exposing the hiPSCs to the FBR. Thereafter, the suspended cells were recirculated between the MCV and FBR at a $Q$ of 60–360 mL min^−1^ to support cell attachment and distribution within the FBR. During this time, regular samples of the medium in the recirculation loop were taken to determine $VCD$ over $t$ until an equilibrium ($VC{D}_{eq}$) was reached after 2–6 h. To accommodate the interpretation of the results, the rate of attachment (${k}_{a}$) and detachment (${k}_{d}$) were approximated by assuming a simple reversible first-order reaction (Atkins and de Paula 2006) and by accounting for the cell density at inoculation ($VC{D}_{0}$) alongside $VC{D}_{eq}$, as shown in Eq. (5).

5 $$VCD(t)=\left(VC{D}_{0}-VC{D}_{eq}\right)\times {e}^{-\left({k}_{a}+{k}_{d}\right)\times t}+VC{D}_{eq}$$

Furthermore, given that attachment efficiency ($AE$) at $t$ could not be observed within the FBR, it was indirectly determined by quantifying $VCD(t)$ within the supernatant at regular intervals and comparing it to the $VC{D}_{0}$, as shown in Eq. (6).

6 $$AE(t)=\frac{VC{D}_{0}-VCD(t)}{VC{D}_{0}}$$

Following the attachment phase, the cells were cultivated for 24 h in batch mode, followed by a 24 h fed-batch phase, where ${V}_{L}$ was increased to 2 L and a $V/A$ of ≈ 0.2 mL cm^−2^ at a flow rate of ≈ 0.9 mL min^−1^ (125–130 µL cm^−2^ day^−1^). Following the completion of the fed-batch phase, fresh medium was perfused at a $D$ of 0.9–2.8 day^−1^ (185–555 μL cm^−2^ day^−1^) for 2–5 days. During this time, two different DO control strategies were used. DO within the MCV was either regulated to minimize $Q$ between MCV and FBR, restricting hiPSC exposure to $\tau$, or the DO gradient in the FBR was restricted to ≤ 20% by regulating $Q$.

Cell harvest was performed 5 days post-inoculation to quantify cell growth and quality. Cell distribution ($CD$) and maximum $HE$ were determined directly before harvest by removing and exposing individual discs from the top, middle, and bottom of the FBR to proteolytic reagent or crystal violet staining solution. Next, the entire FBR was either completely drained and directly exposed to the harvest solution or washed with PBS and then exposed to the harvest solution for 30–40 min at 20–37 °C. During this time, to support cell detachment before redirection to the collection vessel, the harvest solution was recirculated through the FBR at a $Q$ of either 60–500 mL min^−1^ using the recommended harvest kit or at 240–960 mL min^−1^ using a custom-built harvest loop. Towards the end of the harvest procedure, cell detachment, collection, and harvest solution quenching were facilitated by flushing the FBR with a combination of RI-supplemented cultivation medium and compressed air at an overpressure of 0.8 bar.

### Analytical techniques

#### Evaluation of confluence and cell distribution

Images were taken using the EVOS™ FL 2 Auto (Thermo Fisher Scientific Inc., US) or Dmi1 (Leica, DE) microscopes. Similar to the method reported by Lambrechts et al. (2016b), changes to confluence during the XP10 experiments were monitored using the Dino-Lite Digital Microscope (AnMo Electronics Corporation, TW). In all cases, image segmentation was performed using ilastik v1.4 (University of Heidelberg, DE) following image acquisition, with further post-processing carried out using Matlab 2022a (Mathworks, US), as previously described by Teale et al. (2024).

Alongside label-free monitoring of cell growth, cells were stained to allow macroscopic changes in cell confluence and distribution to be observed on opaque scaffolds or surfaces. This was achieved by treating the cells with 10% neutral buffered formalin and then staining them with a 5 g L^−1^ crystal violet solution. The staining solution was prepared by dissolving crystal violet powder (Merck, DE) in an aqueous 200 mL L^−1^ methanol (Merck, DE) solution. Prior to image acquisition, the fixed and stained cells were washed up to ten times with PBS to remove any unbound staining reagent.

#### Evaluation of cell count and viability

As described in more detail by Schneider et al. (2025), $VCD$ and viability were determined following single cell harvest using either the NucleoCounter^®^ NC-200™ and Via1-Cassettes™ or NC-202™ and Via2-Cassettes™ (ChemoMetec, DK), respectively. Alongside the measurement of $VCD$, doubling times (${t}_{d}$), $EF$ and $HE$, were quantified using established techniques described in greater detail elsewhere (Narumi et al. 2020; Teale et al. 2024).

#### Medium component analysis

Indirect monitoring of cell growth and death was achieved through daily bioreactor sampling and analysis of medium component concentrations or enzymatic activity within the supernatant. Sample analysis was conducted using the Cedex^®^ Bio (Roche, US) and corresponding reagent kits for glucose (Glc), glutamine (Gln), lactate (Lac), ammonium (NH4), and lactate dehydrogenase (LDH). Together with the observed changes to cell density, these component concentrations allowed for the calculation of cell-specific consumption and production rates (${q}_{s}$), alongside their respective yields (${Y}_{A/B}$), as described in greater detail by Teale et al. (2024).

#### Analysis of cell identity and potency

Prior to inoculation and following harvest, hiPSC identity and differentiation potential were determined as described by Teale et al. (2024). Briefly, for identity, the expression of pre-selected pluripotency markers Oct3/4, Sox2, Nanog, TRA-1–60, and SSEA-4 and the differentiation marker SSEA-1 were quantified in > 10^4^ cells using the MACSQuant^®^ 10 (Miltenyi Biotec, DE) flow cytometer (FCM) and suitable fluorophore-conjugated antibodies (Miltenyi Biotec, DE and BioLegend, US). In parallel, single cells were plated on rhVTN-coated TC-treated 6-well plates and brought to differentiate towards either an endo-, meso-, or ectodermal lineage over 5–7 days using the STEMdiff™ Trilineage Differentiation Kit (STEMCELL Technologies, CA) to determine potency. Successful differentiation was confirmed following single cell harvest, staining, and FCM analysis by quantifying marker combinations typical for either endo- (Sox17^+^/CD184^+^), meso- (CD56^+^/CD184^+^), and ectodermal (Nestin^+^/Pax6^+^) tissue in > 10^4^ cells. Cells were treated with the Transcription Factor Staining Buffer Set (Miltenyi Biotec, DE) prior to staining all intracellular markers.

## Results

### Numerical and experimental characterization

**Fig. 4:**
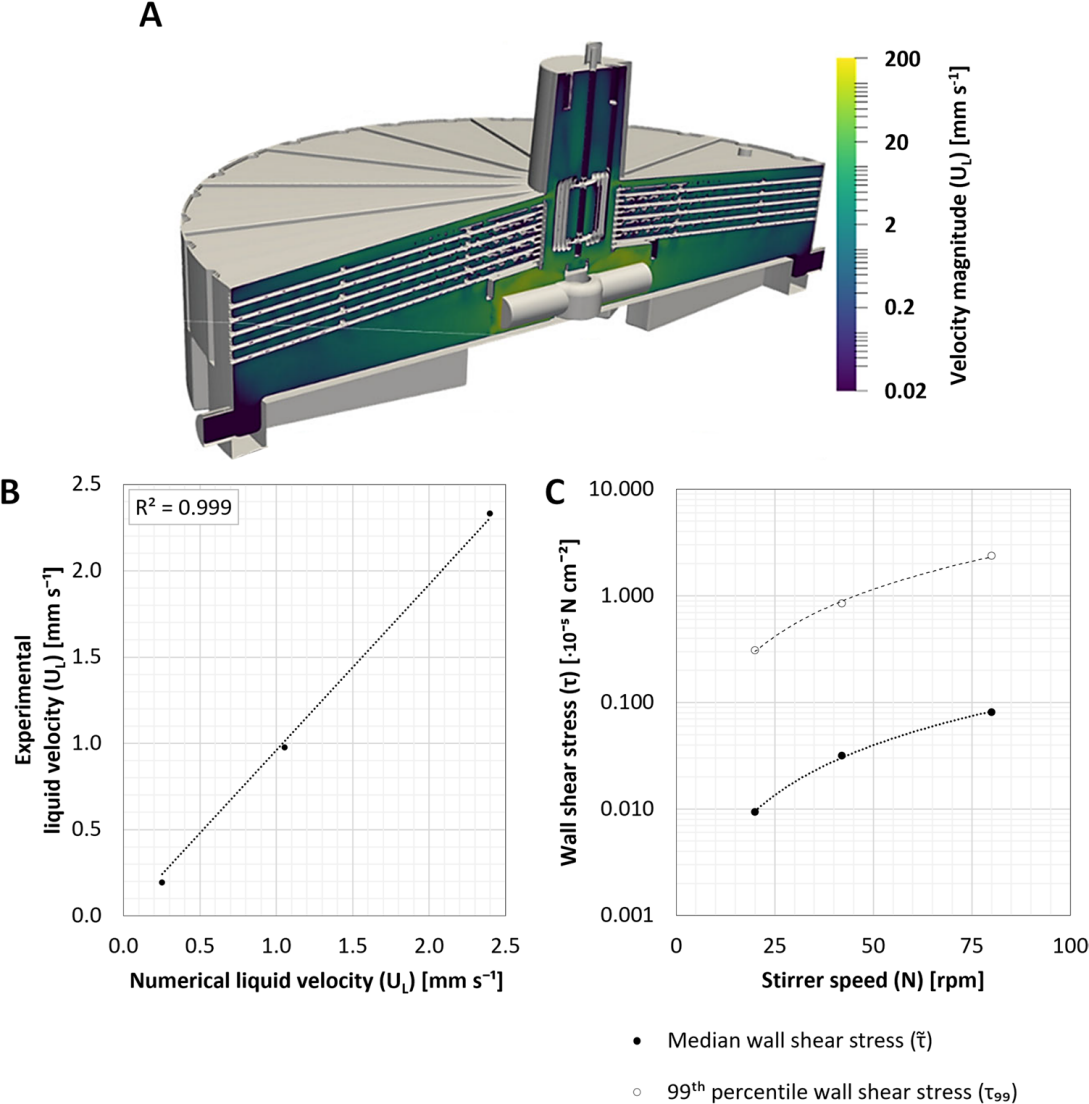
$N$-dependent ${U}_{L}$ profile within the XP10 and resulting $\tau$. **A** ${U}_{L}$ profile within the XP10 bioreactor at the maximum ${V}_{L}$ while operated at a $N$ of 42 rpm. **B** Comparison of the experimentally determined ${U}_{L}$ values, as reported by Yablonsky et al. (2021), to those numerically determined using CFD at identical $N$. **C** $N$-dependent $\widetilde{\tau }$ and ${\tau }_{99}$ wall shear stress acting on the upward facing surfaces of the bioreactors treated plates

The numerical analyses of the XP10 focused on the ${U}_{L}$ profile between the bioreactor’s plates (Fig. 4A), which, given its design, was assumed to be a function of $N$. This was assessed by selecting three different $N$ for in silico investigation, with the median $N$ based on recommendations made by the manufacturer. These investigations confirmed that although a maximum ${U}_{L}$ of ≈318 mm s^−1^ was determined directly adjacent to the magnetic stir bar, the ${U}_{L}$ profile between the plates remained almost constant at 0.25–2.40 mm s^−1^, correlating linearly to $N$ when operated between 20 and 80 rpm (Fig. 4A). Within this range, the ${U}_{L}$ between the plates compared well with the experimentally determined values published by the manufacturer (Yablonsky et al. 2021), validating the results of the numerical model (Fig. 4B). Further evaluation of the upwards-facing surfaces of the XP10’s plates, where cell attachment was likeliest, demonstrated the dependency of $\tau$ to $N$, with a median ($\widetilde{\tau }$) and 99^th^ percentile wall shear stress (${\tau }_{99}$) of 0.01–0.08 × 10^−5^ N cm^−2^ and 0.33–2.52 × 10^−5^ N cm^−2^, respectively, calculated for the evaluated operating range (Fig. 4C).

Further restriction of the operating space to a $N$ to 42–80 rpm and a ${F}_{c}$ of 60–140 mL min^−1^, permitted a ${\theta }_{M}$ of between 17.5 and 64.6 min (Yablonsky et al. 2021) and a ${k}_{L}a$ of 0.03–0.04 h^−1^ to be realized, while limiting $\widetilde{\tau }$ to < 0.08 × 10^−5^ N cm^−2^. Given the notably high ${\theta }_{M}$ reported under these conditions, $\overline{t }$ was additionally assessed at a $N$ of 42 rpm to confirm sufficient mixing within the XP10 when operated in perfusion mode. Ideally, when perfused at a $D$ of ≈ 0.7 day^−1^, this would result in a respective $\overline{t }$ and a ${\sigma }^{2}$ of ≈ 1.35 day and ≈ 0.88 day^2^ (Paul et al. 2004; Fogler 2016). Comparatively, during experimental testing, the XP10 achieved a $\overline{t }$ of 1.39 ± 0.04 day and a ${\sigma }^{2}$ of 0.85 ± 0.03 day^2^, thereby confirming near ideal tracer distribution within the XP10 under these conditions.

**Fig. 5:**
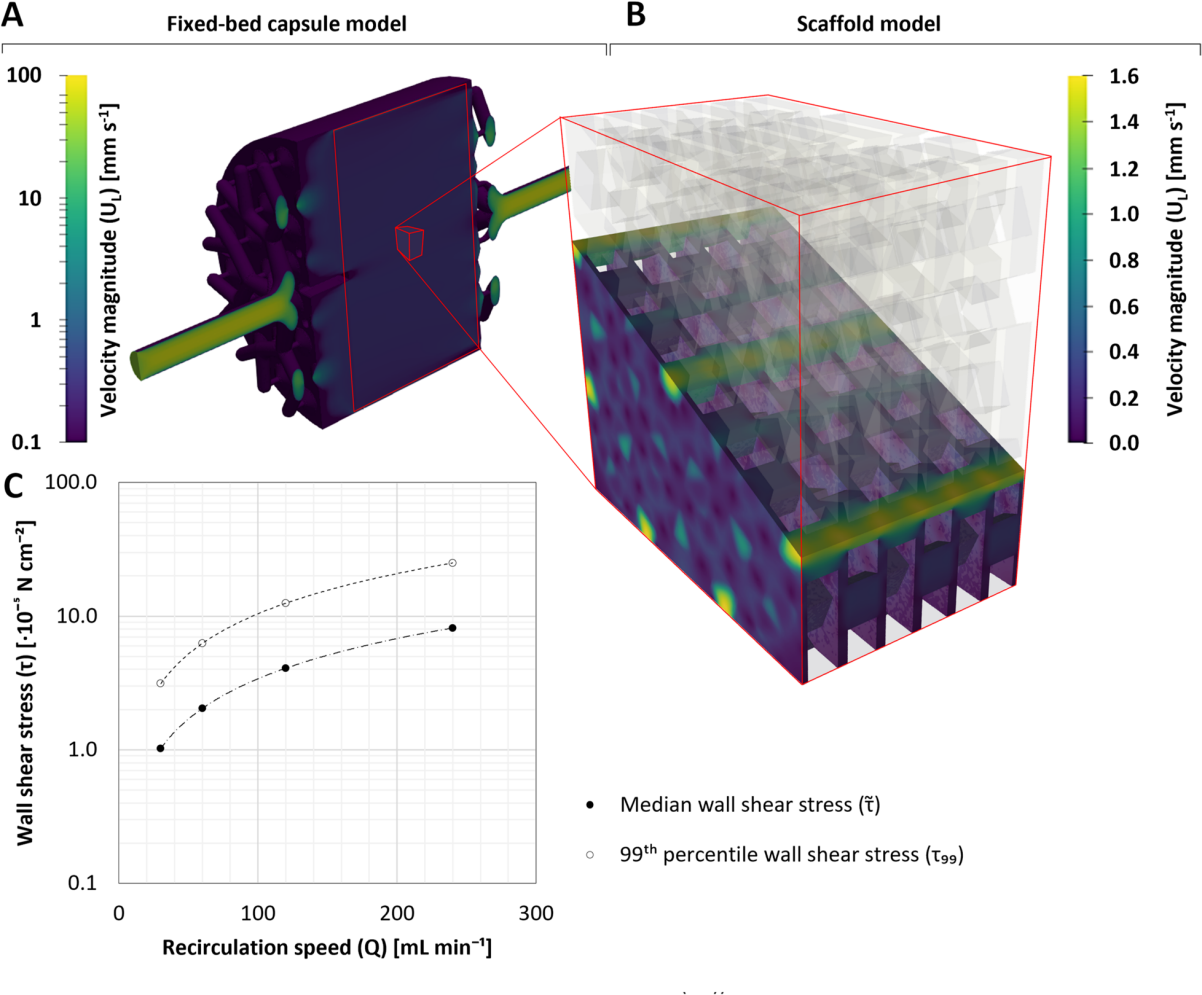
$Q$-dependent ${U}_{L}$ profile and resulting $\tau$ within the FBR of the AS1. **A** ${U}_{L}$ profile in the AS1’s FBR, where the scaffold was simulated as a porous zone and exposed to a $Q$ of 60 ml min^−1^. **B** A more detailed simulation of ${U}_{L}$ profile within the fixed-bed scaffold at 60 mL min^−1^. **C** $Q$-dependent $\widetilde{\tau }$ and ${\tau }_{99}$ acting on the cells within the scaffold

For the AS1, CFD investigations were limited to the bioreactors FBR compartment. Modeling the entire FBR with the scaffold as a porous zone (assuming a Darcy coefficient of 6.85 × 10^9^ m^−2^ in Y-direction) demonstrated that ${U}_{L}$ could be considered homogeneous over the entire cross-section, achieving this state almost immediately after entering the compartment (Fig. 5A). These observations were attributed to the AS1’s branching system, which, in the absence of blockages, promoted uniform fluid flow within the FBR upon entry when operating at a $Q$ of 30–240 mL min^−1^.

The shortfall of the macroscopic simulation, namely that no statement could be made about the prevailing $\tau$ within the scaffold, was resolved by simulating a 2 mm × 2 mm section of the scaffold in greater detail. For this microscopic simulation, cyclic boundary conditions were selected for the inlet and outlet (Fig. 5B), whereby the fixed mean ${U}_{L}$ values determined during the initial macroscopic simulations were used. Given that visual examinations confirmed that all scaffold surfaces may be occupied by hiPSCs (Figure S1), the $\tau$ of the entire available surface area was evaluated. The results of the microscopic model demonstrated that, similarly to the XP10, $\tau$ correlated with $Q$ (Fig. 5C), resulting in a $\widetilde{\tau }$ and ${\tau }_{99}$ of 1.02–8.16 × 10^−5^ N cm^−2^ and 3.12–25.00 × 10^−5^ N cm^−2^, respectively, when operated at 30–240 mL min^−1^. Parallel to the numerical simulations, experimental evaluations of the MCV further demonstrated that a ${\theta }_{M}$ of 0.02–12 min and ${k}_{L}a$ of 0.10–4.73 h^−1^ were achievable when operated at a $N$ of 40–400 rpm, a ${F}_{c}$ of 5–350 mL min^−1^, and a ${V}_{L}$ of 500–3500 mL.

#### Biological evaluation of the Xpansion^®^ 10 multiplate bioreactor

**Fig. 6:**
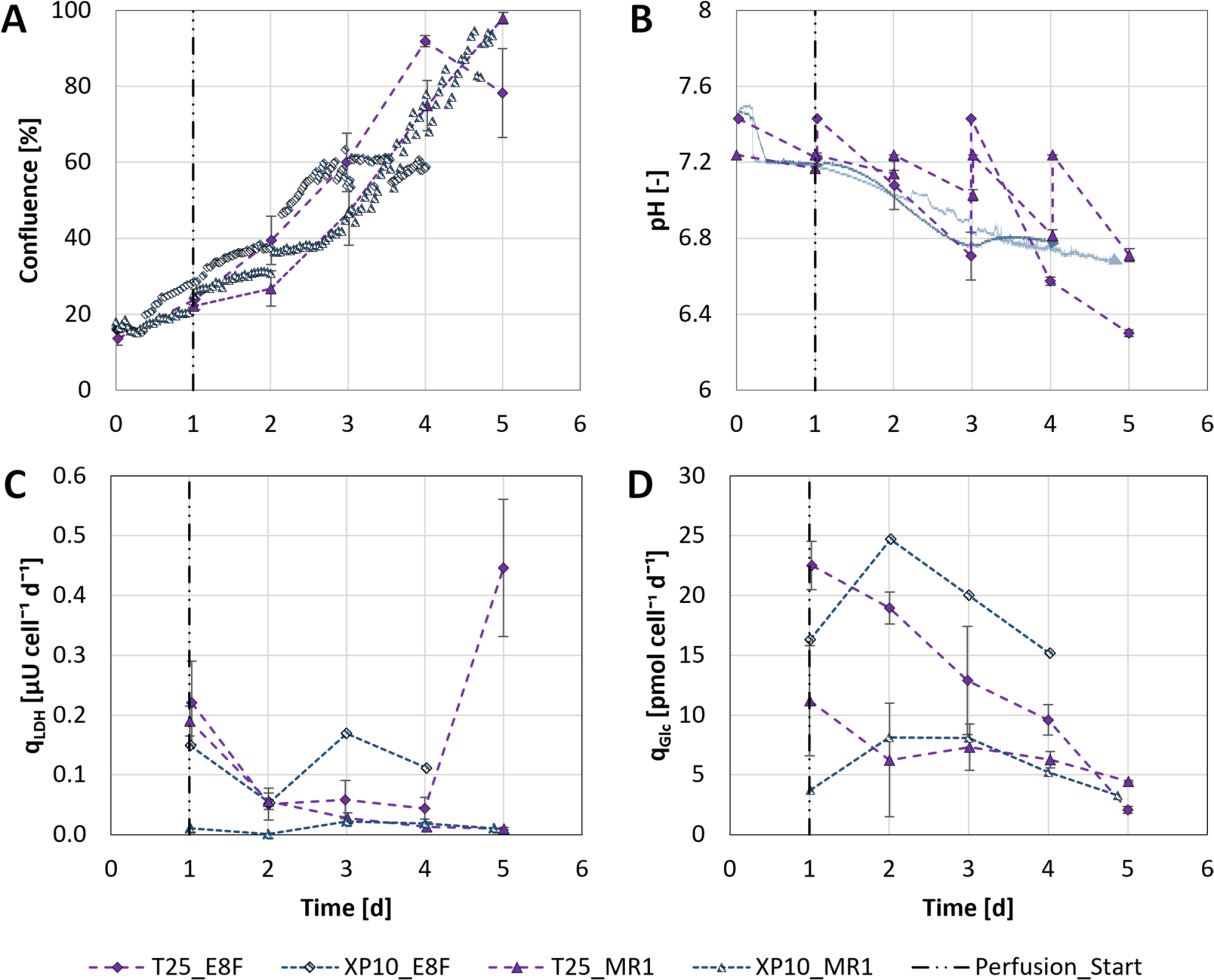
Cell growth and metabolism within the XP10 and T-flask controls. **A** Change in confluence on the XP10’s topmost plate and the base of the T-Flask controls compared to changes in **B** pH process values over the cultivation period. **C** ${q}_{LDH}$ and **D** ${q}_{Glc}$, both of which served as indirect indicators of hiPSC viability and growth. The vertical line on day 1 signifies the start of perfusion mode operation

The suitability of the selected operating range for hiPSC cultivation was demonstrated by inoculating the rhVTN-coated XP10s with cells previously expanded in either E8F or MR1 and possessing a respective viability of 93.6 ± 3.8% and 99.0 ± 1.1%. While no significant cell detachment could be observed microscopically following the 4 h attachment phase and initialization of the control loops (Fig. 6A), pH (Fig. 6B) and DO required up to 12 h to reach their designated setpoints. Maintaining these setpoints proved even more challenging, especially in the case of pH, with gradual acidification of the cultivation medium already noted 1 day following inoculation, irrespective of composition and gassing strategy. A more detailed analysis of the spent medium revealed that both CO_2_ and Lac accumulation were responsible for the observed phenomenon.

To promote the dilution of RI and delay acidification, $D$ was adjusted to 0.7 day^−1^ on day 1. This proved only partially successful in producing the desired effect, however, with pH falling below 6.8 on day 3 during the E8F run (Fig. 6B). As a result, cell growth became stationary at a confluence of ≈ 60% (Fig. 6A) and ${q}_{LDH}$ increased to > 0.15 µU cell^−1^ day^−1^ (Fig. 6C) between days 3 and 4, even after $D$ was adjusted to 1.4 day^−1^. To account for this during the MR1 run, $D$ was adjusted to 1.4 day^−1^ a day earlier, delaying the pH from reaching 6.8 by > 12 h (Fig. 6B). Although exceeding a confluence ≈ 80% caused DO to fall to 15% after day 4, this approach not only produced a significantly higher final confluence of ≈ 90% (Fig. 6A) but also kept ${q}_{LDH}$ below 0.05 µU cell^−1^ day^−1^ (Fig. 6C) throughout the expansion process. Furthermore, alongside the desired effects, such $D$ ensured that Glc and Gln concentrations were kept above 5.6 mmol L^−1^ and 1.0 mmol L^−1^, while Lac and NH4 concentrations remained below 11.0 mmol L^−1^ and 1.5 mmol L^−1^, producing comparable ${q}_{Glc}$ trends to those observed for the respective T-flask controls (Fig. 6D).

**Fig. 7:**
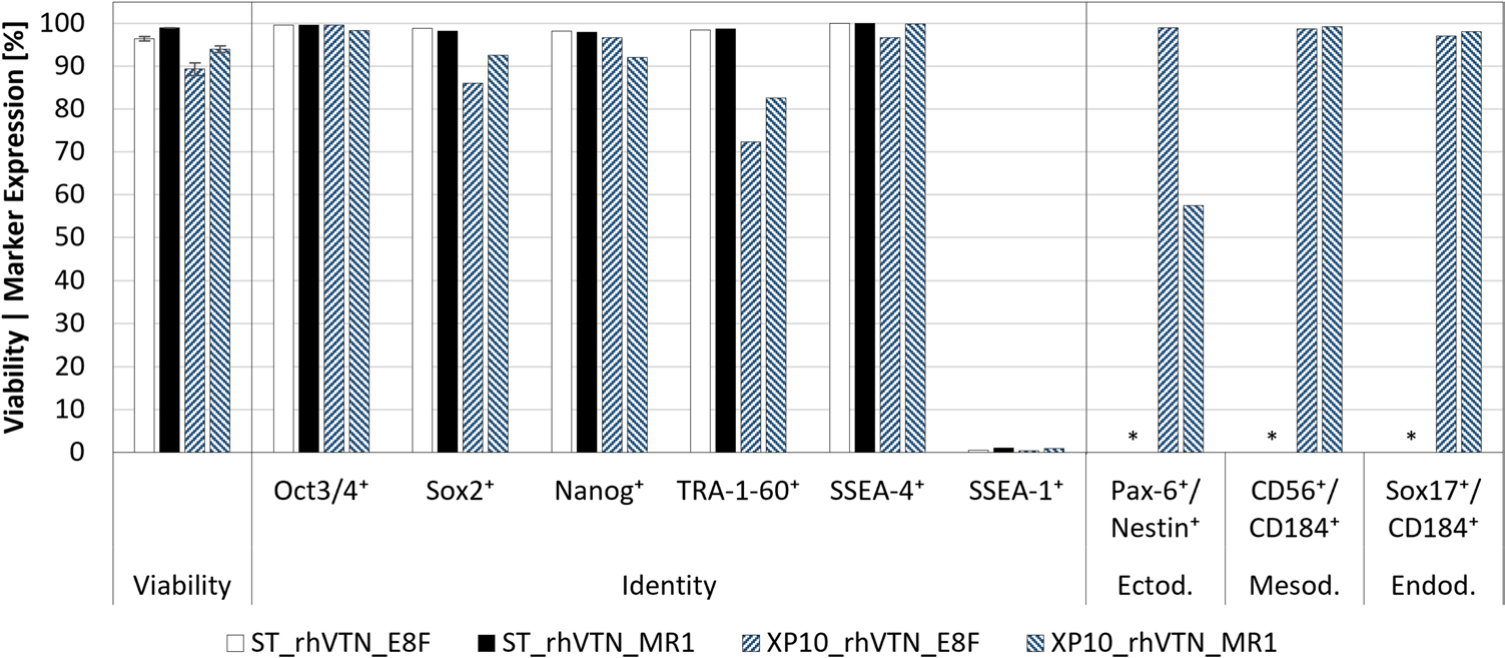
Comparison of hiPSC quality prior to and following serum-free expansion in rhVTN-coated XP10s. Cell viability and identity were quantified following bioreactor harvest, with potency determined independently by differentiating the harvested cells towards the endo-, meso-, and ectodermal germ layers over 5–7 days. In all cases, marker expression was quantified by FCM. *Data unavailable

Both XP10s were harvested 4–5 days post-inoculation to quantify hiPSC yield and quality. Based on the confluence estimates directly prior to harvest, application of the described harvesting method resulted in a $HE$ of 101 ± 6% and 102 ± 6% for the E8F and MR1 runs, yielding 0.8 ± 0.0 × 10^9^ and 4.1 ± 0.3 × 10^9^ viable cells at an $EF$ of 6.0 ± 0.2 and 35.3 ± 2.4, respectively. Given that a $HE$ of > 100% is theoretically impossible, it must be stated that in both experiments, cell confluence was only monitored for a single position on the XP10’s topmost plate. Analysis of the harvested hiPSCs (Fig. 7) demonstrated that viability could be maintained, with 89.3 ± 1.8% and 94.0 ± 0.9% of the cells cultivated in E8F and MR1, respectively, demonstrating membrane integrity following expansion and harvest. FCM analyses further revealed that > 70% of the cell population expressed all pluripotency markers, while > 50% expressed all relevant germ layer-specific marker combinations following differentiation.

#### Biological evaluation of the Ascent™ 1-m^2^ fixed-bed reactor

**Fig. 8:**
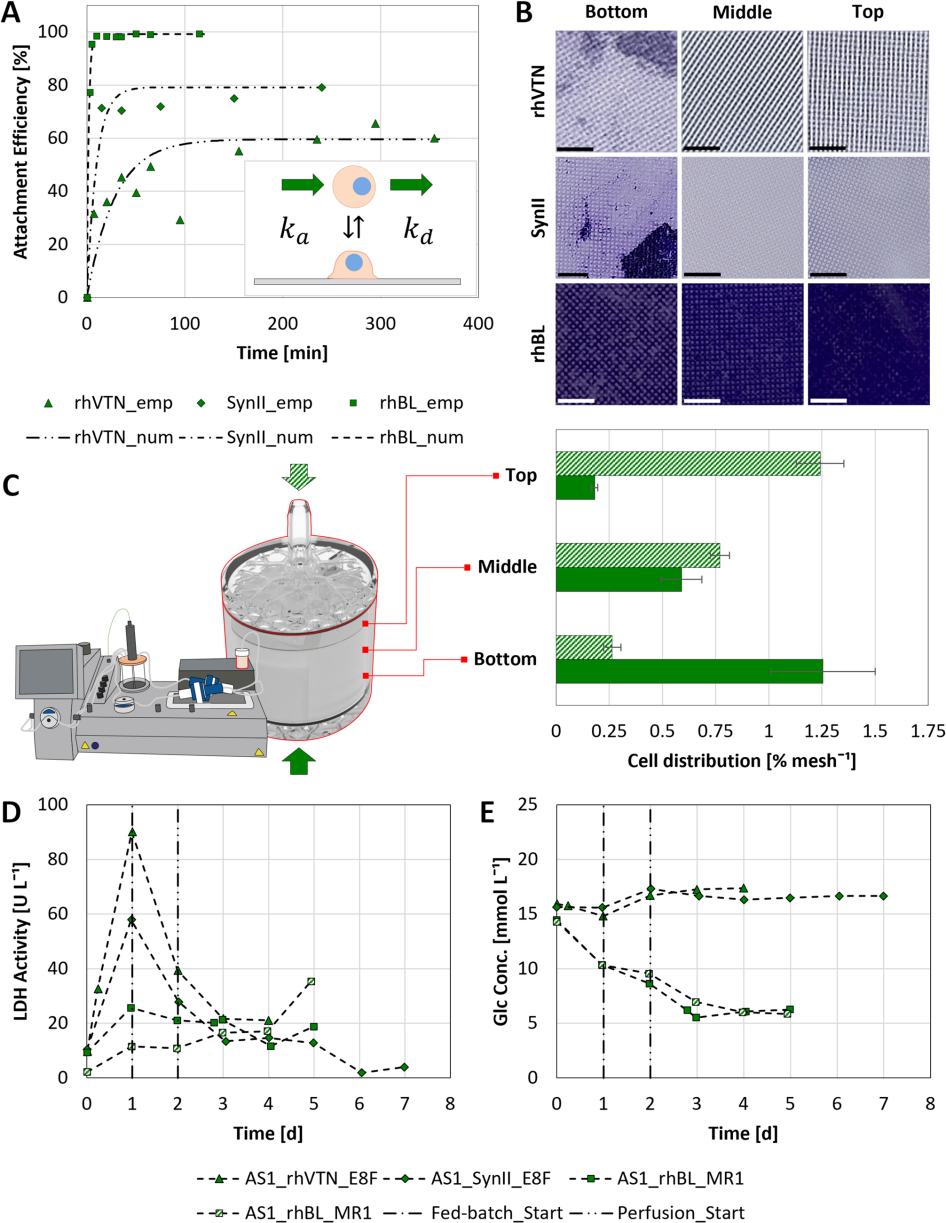
Cell attachment and growth within the AS1. **A** Coating-dependent attachment kinetics during the attachment phase. **B** Qualitative and **C** quantitative $CD$ within the rhBL-coated FBR prior to harvest, depending on the direction of recirculation during the attachment phase. The scale bar in the lower left corner corresponds to 3 mm. Influence of coating choice on **D** LDH activity and **E** Glc metabolism during cultivation

Following the coating of the AS1’s FBR with either rhVTN, SynII, or rhBL, hiPSCs expanded in either E8F or MR1 with respective viabilities of 97.1 ± 0.9% or 97.5 ± 1.4% were used to inoculate the AS1. Subsequent evaluation of $AE$ over $t$ during the attachment phase indicated that neither rhVTN nor SynII was sufficiently potent to secure complete cell attachment after > 4 h when operated at a $Q$ of 60 mL min^−1^ (Fig. 8A). On the contrary, hiPSC attachment to the rhBL-coated FBR at $Q$ of up to 360 mL min^−1^ was almost instantaneous, yielding similar, recirculation direction-dependent $CD$ prior to harvest (Fig. 8B, C). Further evaluation of extracellular LDH activity following inoculation (Fig. 8D) corroborated these findings, with a notable increase to 90 U L^−1^ and 60 U L^−1^ measured within the first 24 h for the rhVTN and SynII-coated AS1s, respectively, suggesting significant cell death. Analyses of Glc in the supernatant over 4–7 days (Fig. 8E), alongside the removal and staining of scaffold sections from the FBR with crystal violet prior to harvest (Fig. 8B), finally concluded a distinct lack of metabolic and proliferative activity for both coatings. On the contrary, following hiPSC attachment to the rhBL-treated FBR, LDH activity remained below 25 U L^−1^ (Fig. 8D), only slightly increasing after 4 days as the discs at the outer edges of the FBR became confluent (Fig. 8B). Alongside rapid attachment and low LDH activity, the hiPSCs attached to the rhBL-coated AS1 also demonstrated significant metabolic activity, as may be surmised from the changes to Glc concentration over the 5-day cultivation period (Fig. 8E).

As with the XP10, the application of perfusion mode operation allowed respective Glc and Gln concentrations to be kept above 5.5 mmol L^−1^ and 0.9 mmol L^−1^, while Lac and NH4 concentrations were kept below 15 mmol L^−1^ and 1.8 mmol L^−1^ during cultivation. However, unlike the XP10, the design of the AS1 allowed for superior pH and DO control, ensuring that the specified setpoints were achieved within minutes of initiating the corresponding control loops. Furthermore, when operated in tandem with perfusion mode, the MCV’s sparger facilitated effective CO_2_ stripping, keeping pH > 6.8. Alongside pH, sparging sustainably restricted the DO gradient within the FBR to ≤ 20% or ≤ 60%, depending on the control strategy employed. In this manner, superior process control during operation kept the impact on cell growth, metabolism, and viability to a minimum, with extracellular LDH activity remaining < 40 U L^−1^ (< 0.05 µU cell^−1^ day^−1^) throughout both runs (Fig. 8C).

**Fig. 9:**
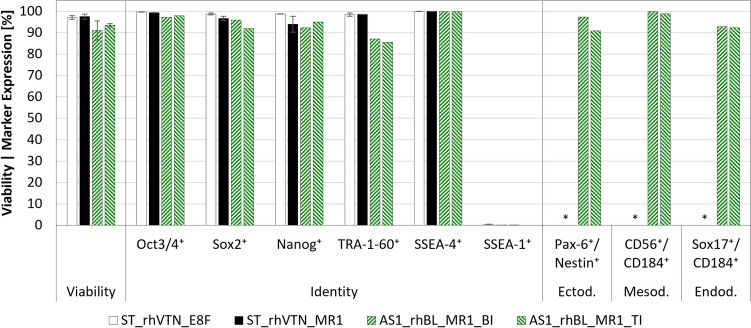
Comparison of hiPSC quality prior to and following serum-free expansion in the rhBL-coated AS1. Cell viability and identity were quantified prior to inoculation (ST) and following harvest of the bottom- (BI) or top-inoculated (TI) FBRs. hiPSC potency was demonstrated through independent differentiation towards the endo-, meso-, and ectodermal germ layers over 5–7 days. In all cases, marker expression was quantified by FCM. *Data unavailable

As with the XP10, cell growth within the FBR was quantified by harvesting both AS1s after 5 days, yielding similar viable cell quantities of 4.0–4.6 × 10^9^. Remarkably, performing the harvest at 37 °C and lower $Q$, as opposed to 20–25 °C and higher $Q$, was shown to improve $HE$ from 81.4 ± 7.6% to 94.3 ± 6.1%, without impacting cell viability, identity, or differentiation potential (Fig. 9). Here, as with the XP10 trials, cell viability exceeded the recommended minimum of 70% (Sullivan et al. 2018) with 91 ± 4.8% and 93.5 ± 0.8% for the first and second MR1 runs, respectively. Subsequent analysis by FCM demonstrated that all pluripotent markers were present in > 85% of the population, while differentiation to the three germ layers confirmed cell potency, with the associated marker combinations present in > 90% of the differentiated cells.

## Discussion

The purpose of the current study was to evaluate whether, following adequate characterization and designation of a suitable operating range, the XP10 and AS1 could support the production of hiPSCs at L-scale under serum-free conditions. This could clearly be proven, with both bioreactors achieving similar results to those reported for the microcarrier-operated dual-impeller BioBLU^®^ 1c [BB1] (Schneider et al. 2025) within the same time (Table 1) without significant loss of viability, identity, or differentiation potential.

**Table 1:** Comparison of hiPSC growth and quality in perfusion mode operated SU bioreactors based on the prevailing bioengineering parameters during cultivation

Parameter	Xpansion^®^ 10		Ascent™ 1 m^2^	BioBLU^®^ 1c dual-impeller	
${V}_{L}/A$ [mL cm^−2^]	≈0.26		0.07–0.20	0.09–0.28	
$N$ [rpm] or $Q$ [mL min^−1^]*	42–80		60–240*	57	
${U}_{L}$ [mm s^−1^]	1–2.3		0.7–2.6	-	
$\widetilde{\tau }$ [× 10^−5^ N cm^−2^]**	0.03–0.08		2.04–8.16	0.23–0.27	
${\tau }_{99}$ [× 10^−5^ N cm^−2^]**	0.89–2.31		6.25–25.0	4.82–5.46	
${k}_{L}a$ [h^−1^]	0.03–0.04		0.35–2.95	1.00–3.81	
${\theta }_{M}$ [min]	17.5–64.6		< 1.5	0.2–2.2	
$\overline{t }$(${\sigma }^{2}$) [day (day^2^)]	1.39 ± 0.04 (0.85 ± 0.03)		-	-	
Medium	E8F	MR1	MR1	E8F	MR1
Scaffold	Polystyrene		PET	Polystyrene	
Coating	rhVTN		rhBL	SynII	
Attachment phase [h]	4		2	12	6–12
${q}_{Glc}$ [pmol cell^−1^ day^−1^]	19.1 ± 4.3	5.7 ± 2.3	9.4 ± 3.0	18.0 ± 5.7	6.1 ± 2.4
${q}_{Gln}$ [pmol cell^−1^ day^−1^]	2.6 ± 0.7	1.3 ± 0.4	1.3 ± 0.4	2.7 ± 1.0	0.9 ± 0.4
${q}_{Lac}$ [pmol cell^−1^ day^−1^]	30.8 ± 10.2	10.3 ± 4.3	15.1 ± 5.0	30.7 ± 9.1	10.7 ± 5.7
${q}_{NH4}$ [pmol cell^−1^ day^−1^]	2.1 ± 0.7	0.8 ± 0.2	0.9 ± 0.4	1.8 ± 0.7	0.6 ± 0.3
${q}_{O_2}$ [pmol cell^−1^ h^−1^]	0.013 ± 0.006	0.024 ± 0.005	0.229 ± 0.103	0.443 ± 0.162	0.292 ± 0.082
${Y}_{Lac/Glc}$ [mol mol^−1^]	1.6 ± 0.3	1.8 ± 0.1	1.6 ± 0.1	1.7 ± 0.2	1.7 ± 0.2
${Y}_{NH4/Gln}$ [mol mol^−1^]	0.8 ± 0.1	0.6 ± 0.1	0.7 ± 0.1	0.7 ± 0.1	0.7 ± 0.2
Max. viable cell yield [cells]	0.8 ± 0.0 × 10^9^	4.1 ± 0.3 × 10^9^	4.6 ± 0.3 × 10^9^	1.7 ± 0.1 × 10^9^	3.3 ± 0.2 × 10^9^
Max. $EF$ [-]	6.0 ± 0.2	35.3 ± 2.4	19.5 ± 1.8	21.5 ± 1.0	25.5 ± 1.3
Cultivation time [day]	4	5	5	4–5	5
Min. ${t}_{d}$ [h]	37.5 ± 0.0	23.6 ± 0.1	22.6 ± 0.1	11.1 ± 0.4	11.9 ± 0.4
Max. $HE$ [%]	100.5 ± 5.7	102.4 ± 6.4	94.3 ± 6.1	92.8 ± 10.9	85.9 ± 1.7
Viability [%]	89.3 ± 1.8	94.0 ± 0.9	93.5 ± 0.8	95.7 ± 1.9	95.8 ± 3.0
Pluripotent	Yes	Yes	Yes	Yes	Yes
Tri-lineage differentiation	Yes	Yes	Yes	Yes	Yes

*During the experiments, $N$ was regulated for the Xpansion^®^ 10 and BioBLU^®^ 1c dual-impeller, while $Q$ was regulated for the Ascent™ 1 m^2^. **The numerically simulated wall shear stress for the Xpansion^®^ 10 and Ascent™ 1 m^2^ and shear stress for the BioBLU^®^ 1c dual-impeller

Characterizing the XP10 and AS1 prior to cultivation, using both numerical and experimental bioengineering methods, enabled the $N$ of the XP10’s stirrer bar and the $Q$ of the AS1’s recirculation pump to be correlated to $\tau$ exposure during operation. In this way, $N$ and $Q$ could be controlled to ensure $\widetilde{\tau }$ would remain within a range considered suitable for pluripotent stem cell expansion (Cormier et al. 2006) and below the respective thresholds 10 × 10^−5^ N cm^−2^ and 100 × 10^−5^ N cm^−2^ associated with differentiation (Huang et al. 2021) and cell detachment (Fuhrmann and Engler 2015).

Restrictions placed on $\tau$ naturally impacted other parameters, such as ${k}_{L}a$ and ${\theta }_{M}$. As such, when operating within the established range, the XP10 was limited to a ${k}_{L}a$ of 0.04 h^−1^ and relatively high ${\theta }_{M}$ of ≈ 64 min, although it did demonstrate a near-ideal RTD profile when operated in perfusion mode. In contrast, confinement of the hiPSCs to the AS1’s FBR during cultivation meant that the bioreactors MCV could be sparged and operated at higher $N$ without influencing $\tau$, permitting ${\theta }_{M}$ of < 1.53 min and ${k}_{L}a$ of up to 2.95 h^−1^. When accounting for the ${q}_{O_2}$ of 0.01–0.07 pmol cell^−1^ day^−1^ reported by Abecasis et al. (2017), alongside the values determined during the cultivations, such conditions were estimated to support the production of up to ≈ 5.6 × 10^9^ hiPSCs (≈ 9.2 × 10^5^ cells cm^−2^) in the XP10 and ≈ 26.4 × 10^9^ hiPSCs (≈ 2.6 × 10^6^ cells cm^−2^) in the AS1. Considering that the XP10’s plates and the AS1’s PET-scaffolds were observed to reach confluence at between 4.0 and 8.0 × 10^5^ cells cm^−2^ (Teale et al. 2024; Schneider et al. 2025) whether, following adequate characterization and de) depending on the hiPSC phenotyhe purpose of the current study was to evaluate whether, following adequate characterization and de) depending on the hiPSC phenotype, such ${\theta }_{M}$ and ${k}_{L}a$ values were considered acceptable for the adherent expansion of these cells in both bioreactors.

The successful expansion of adherent hiPSCs demands timely attachment to the target scaffold, as failure to do so risks loss of cell quality either through excessive aggregation (Chen et al. 2010a; Kim et al. 2019) or dissociation-induced apoptosis (Watanabe et al. 2007; Kim et al. 2019). In the absence of serum, hiPSC attachment is regulated through the use of CAMs (Miyazaki et al. 2017; Schneider et al. 2025), which act as intermediaries between specific proteins on the cell surface and the scaffold (Rowland et al. 2010). Given the compositional similarity between the XP10’s treated polystyrene plates (Lambrechts et al. 2016b) and conventional TC-treated cultureware (Lerman et al. 2018), it was, therefore, reasonable to assume that rhVTN would prove a potent CAM following exposure of the adherent hiPSCs to a $\widetilde{\tau }$ of 0.03 × 10^−5^ N cm^−2^. In contrast, when applied to the AS1’s PET-based scaffold, equivalent concentrations of rhVTN failed to accommodate meaningful hiPSC attachment at a $\widetilde{\tau }$ of 2.04 × 10^−5^ N cm^−2^, resulting in a low ${k}_{a}$ of 0.02 min^−1^. A second attempt using tenfold higher concentrations of SynII, a synthetic alternative to rhVTN (Martin et al. 2012), likewise led to a low ${k}_{a}$ of 0.05 min^−1^ and incomplete cell attachment. In both instances, this $AE$ of 60–80%, although typical for hiPSCs (Legrand et al. 1992; Paccola Mesquita et al. 2019; Schneider et al. 2025), led to reduced metabolic activity and ultimately cell death. However, when using rhBL, hiPSCs were observed to attach to the scaffold within 5 min at a ${k}_{a}$ of 0.27 min^−1^, producing $AE$ of > 93% at $\widetilde{\tau }$ of up to 12.24 × 10^−5^ N cm^−2^. These results suggest that differences in scaffold properties, particularly between polystyrene and PET-based materials, play a crucial role in determining CAM potency for hiPSC attachment and expansion (Badenes et al. 2016; Miyazaki et al. 2017; Paccola Mesquita et al. 2019).

Alongside rapid attachment to the target scaffold, processes where hiPSCs are adherently cultivated must additionally account for $CD$, given the cells’ inherently low motility (Zhang et al. 2011). Enhancing $CD$ promotes more uniform colony formation, delaying contact inhibition and supporting sustained cell growth (Kim and Kino-oka 2020). Such observations have already been made when expanding hiPSCs in MC-operated stirred bioreactors, where $CD$ was shown to correlate with improved initial cell growth and overall yield (Teale et al. 2024; Schneider et al. 2025). Recognizing the importance of this parameter, spatial analyses of confluence and relative $CD$ were conducted on the FBR’s disc-shaped scaffolds at set intervals during cultivation. These analyses revealed that the direction of recirculation following inoculation had the greatest impact on $CD$, determining which discs were confluent at harvest (≈ 8.0 × 10^5^ cells cm^−2^) and which were not (≈ 1.5 × 10^5^ cells cm^−2^) with a sevenfold difference noted between discs at the FBR’s inflow and outflow. Consequently, although inoculation cell densities were kept similar between the AS1 and XP10, discs at the FBR’s inflow reached confluence earlier, limiting the final $EF$ of the AS1 to 19.5 ± 1.8 compared to the XP10’s 35.3 ± 2.4 after 5 days (Table 1).

Closer evaluation of metabolic activity revealed that the hiPSCs cultivated in E8F displayed threefold higher ${q}_{Glc}$ and twofold higher ${q}_{Gln}$, yet similar ${Y}_{Lac/Glc}$ and ${Y}_{NH4/Gln}$, when compared to those cultivated in MR1 (Table 1). In all instances, metabolic activity peaked directly following RI dilution, gradually decreasing towards the end of cultivation by a factor of 2–4 and reflecting what has been observed when cultivating these cells as spheroids at smaller scales (Manstein et al. 2021; Ullmann et al. 2024). Given that nutrient concentrations remained abundant, the decline in activity was attributed to cell quiescence resulting from contact inhibition (Kim and Kino-oka 2020; Marescal and Cheeseman 2020), with poor pH regulation in the E8F-operated XP10 proving the singular exception. Consistent with previous studies (Teslaa and Teitell 2015; Zhang et al. 2018; Horiguchi and Kino-oka 2021), the hiPSCs initially favored aerobic glycolysis over oxidative phosphorylation, with a ${Y}_{Lac/Glc}$ of up to 1.9 mol mol^−1^ measured over 2 days following inoculation of the XP10 and AS1. However, following the dilution of RI, a slight drop in the specific growth rate and a metabolic shift towards oxidative phosphorylation was noted. This shift was accompanied by a slight decrease of the ${q}_{O_2}$ to ≈ 0.01 pmol cell^−1^ h^−1^ during the E8F experiments and an increase to between 0.02 and 0.23 pmol cell^−1^ h^−1^ during the MR1 experiments, falling well within the reported range of 0.01–0.31 pmol cell^−1^ h^−1^ (Abecasis et al. 2017; Greuel et al. 2019). As inhibitory concentrations of Lac and NH4 were avoided for the most part during expansion (Chen et al. 2010b; Horiguchi et al. 2018) and as the shift coincided with slightly lower proliferation rates, a lower ${Y}_{Lac/Glc}$, and higher cell respiration during the MR1 experiments, this behavior was attributed to the hiPSCs returning to a more naïve state (Teslaa and Teitell 2015; Zhang et al. 2018; Tsogtbaatar et al. 2020).

Although not as extensive as the analytical panels recommended by Sullivan et al. (2018) and Sebastião et al. (2021), hiPSC viability, identity, and differentiation potential were evaluated following harvest to confirm cell quality and the absence of spontaneous differentiation (Teslaa and Teitell 2015; Zhang et al. 2018). These assays concluded that pluripotent marker expression in the cell populations harvested from the XP10 and AS1 exceeded > 89% and > 70%, respectively, with all maintaining the ability to differentiate into all three germ layers. Furthermore, with SSEA-1 expression consistently ≤ 1%, spontaneous differentiation was effectively mitigated (O’Shea et al. 2020; Watanabe et al. 2020), confirming the suitability of the selected operating ranges. Consistent with observations made for other pluripotent stem cell lines (Chen et al. 2010b; Meng et al. 2017), maintaining a pH > 6.8 during cultivation improved cell growth, viability, and TRA-1–60 marker expression, while a DO range of 15–40% had no noticeable effect on quality (Abecasis et al. 2017; Horiguchi and Kino-oka 2021).

In closing, when accounting for $\tau$, the XP10 and AS1 proved suitable for hiPSC expansion, achieving yields of up to 4.6 × 10^9^ cells within 5 days alongside $EF$ of ≈ 35. these yields exceed the amounts necessary to treat more than four patients for a majority of clinical indications (Scibona and Morbidelli 2019) and improve on the values reported for other hiPSC expansion processes (Kwok et al. 2018; Paccola Mesquita et al. 2019; Pandey et al. 2020; Huang et al. 2020; Manstein et al. 2021; Cohen et al. 2023). In particular, it could be shown that cultivating hiPSCs adherently at L-scale using either the multiplate or fixed-bed bioreactor allowed a threefold higher $EF$ to be achieved within a similar time than when cultivating the hiPSCs as spheroids in stirred bioreactors (Kwok et al. 2018; Huang et al. 2020). Moreover, these bioreactors did not require the encapsulation of the hiPSCs prior to cultivation (Cohen et al. 2023) or the implementation of microcarriers (Pandey et al. 2020), though accounting for $CD$ was essential to improve hiPSC growth relative to the other adherent platforms (Paccola Mesquita et al. 2019; Pandey et al. 2020). With this in mind, the production of safe hiPSC-based cell therapies requires not only efficient and scalable production of clinically relevant hiPSC quantities but also precise, directed differentiation following a brief 1–7-day expansion phase (Yasuda et al. 2018; Laco et al. 2020; Jacobson et al. 2021; Sivalingam et al. 2021; Nogueira et al. 2021; Teale et al. 2023). Whether these systems can reliably support this next critical step in the manufacturing process remains to be confirmed.

## Supplementary Information

Below is the link to the electronic supplementary material.

Supplementary file1 (PDF 317 KB)

## Data Availability

The datasets generated and/or analyzed during the current study and the code used are available from the corresponding author upon reasonable request.

## Abbreviations

*AS1*: Ascent™ 1 m^2^ fixed-bed bioreactor
*BB1*: Dual-impeller BioBLU^®^ 1c bioreactor
*CAM*: Cell adhesion mediator
*CFD*: Computational fluid dynamics
*CT*: Cell therapy
*DO*: Dissolved oxygen
*E8F*: Essential 8™ Flex
*FBR*: Fixed-bed reactor
*FCM*: Flow cytometry
*Glc*: Glucose
*Gln*: Glutamine
*hiPSC*: Human induced pluripotent stem cell
*Lac*: Lactate
*LDH*: Lactate dehydrogenase
*MCV*: Medium conditioning vessel
*MR1*: m TesR1™
*NH4*: Ammonium
*PBS*: Phosphate-buffered saline
*PET*: Polyethylene terephthalate
*rhBL*: Recombinant human laminin-521
*rhVTN*: Recombinant human vitronectin
*RI*: Pan rho-associated, coiled-coil protein kinase inhibitor Y-27632
*RTD*: Residence time distribution
*ST*: Seed train
*SU*: Single-use
*SynII*: Synthemax II
*TC*: Tissue culture
*XP10*: Xpansion^®^ 10 multiplate bioreactor

## Latin symbols

$$AE$$: -, Attachment efficiency
$$CD$$: -, Cell distribution
$$D$$: s^−^^1^, Dilution rate
$$EF$$: -, Expansion factor
$F$: -, Non-dimensional time-dependent F-curve
${F}_{c}$: m^3^ s^−^^1^, Combined gas flow rate
$$HE$$: -, Harvest efficiency
${k}_{L}a$: s^−^^1^, Volumetric mass transfer coefficient
${k}_{a}$: s^−^^1^, Rate of attachment
${k}_{d}$: s^−^^1^, Rate of detachment
$$N$$: s^−^^1^, Stirring speed
$Q$: m^3^ s^−^^1^, Volumetric flow rate
${q}_{s}$: mol cell^−^^1^ s^−^^1^, Cell-specific consumption/production rate of compound s
$$t$$: s, Time
$\overline{t }$: s, Mean residence time
$${t}_{d}$$: s, Doubling time
$${U}_{L}$$: m s^−^^1^, Liquid velocity
$${V}_{L}$$: m^3^, Working volume
${V}_{L}/A$: m, Working volume-to-surface area ratio
$VCD$: cells m^−^^3^, Viable cell density
$VC{D}_{0}$: cells m^−^^3^, Viable cell density in the supernatant at inoculation
$VC{D}_{eq}$: cells m^−^^3^, Viable cell density in the supernatant at equilibrium
$$v$$: m^2^ s^−^^1^, Kinematic viscosity
$y$: m, Distance in the normal direction
${Y}_{A/B}$: mol mol^−^^1^, Yield of A from B

## Greek symbols

$${\theta }_{M}$$: s, Mixing time
${\kappa }_{0}$: kg^−1^ m^−3^ s^3^ A^2^, Initial conductivity within the bioreactor
${\kappa }_{\infty }$: kg^−1^ m^−3^ s^3^ A^2^, Conductivity of the inert tracer solution
${\kappa }_{t}$: kg^−1^ m^−3^ s^3^ A^2^, Conductivity at the outflow port at time t
${\sigma }^{2}$: s^2^, Variance of the mean residence time
$$\rho$$: kg m^3^, Density
$\tau$: N m^−^^2^, Wall shear stress
$\widetilde{\tau }$: N m^−^^2^, Median wall shear stress
${\tau }_{99}$: N m^−^^2^, 99^th^ percentile wall shear stress
