# Supplementary material for: Expansion of induced pluripotent stem cells under consideration of bioengineering aspects: part 2
Source: Appl Microbiol Biotechnol. 2025 Feb 6;109:38. doi: 10.1007/s00253-024-13373-2 (PMC11802622; doi:10.1007/s00253-024-13373-2)
Supplement: Supplementary file 1 [file 253_2024_13373_MOESM1_ESM.pdf]

Expansion of induced pluripotent stem cells under consideration of bioengineering aspects: Part 2 – Supplementary data

Misha Alexander Teale [1], Samuel Lukas Schneider [1], Stefan Seidel [1], Jürgen Krasenbrink [2], Martin Poggel [2], Dieter Eibl [1], Marcos F. Q. Sousa [2], Regine Eibl [1]*

[1] Centre for Biochemical Engineering and Cell Cultivation Techniques, Institute of Chemistry and Biotechnology, Zurich University of Applied Sciences, Grüentalstrasse 14, 8820 Wädenswil, Switzerland. misha.teale@zhaw.ch, https://orcid.org/0000-0003-0599-4115 (M.A.T.), samuel.schneider@zhaw.ch https://orcid.org/0000-0002-2756-408X (S.L.S.), stefan.seidel@zhaw.ch, https://orcid.org/0000-0002-5244-0855, (S.S.), dieter.eibl@zhaw.ch, https://orcid.org/0000-0003-0033-3393 (D.E.), regine.eibl@zhaw.ch, https://orcid.org/0000-0002-1840-8253 (R.E.)

[2] Advanced Manufacturing-Platform Engineering and Support, Bayer AG, Kaiser-Wilhelm-Allee 1, 51373 Leverkusen, Germany. juergen.krasenbrink@bayer.com (J.K.), marcos.sousa1@bayer.com, https://orcid.org/0000-0003-3766-3900 (M.F.Q.S.), martin.poggel@bayer.com (M.P.)

[*] Corresponding author

SUPPLEMENTARY DATA

Scaffold coating study

If hiPSCs do not attach to a CAM-coated scaffold, they may either form spheroids through aggregation (Chen et al. 2010a; Kim et al. 2019) or undergo dissociation-induced apoptosis (Watanabe et al. 2007; Kim et al. 2019). Since it has been demonstrated that scaffold choice and CAM concentration significantly impact cell attachment and growth (Badenes et al. 2016; Miyazaki et al. 2017; Paccola Mesquita et al. 2019; Dias et al. 2022), identifying PET-compatible CAMs should be considered a critical first step in process development with the AS1. Small sterile discs (Corning Inc., US) with identical properties to The FBRs PET-based scaffold were coated with various CAMs and assessed under static conditions to confirm compatibility.

The discs, with a diameter of 29–34 mm and a surface area of between 16–22 cm^2, were individually coated for > 2 h at 20–25 °C using a 3 mL buffered solution containing either 0.5–1 µg cm^{-2} rhVTN, 5.3–10.5 µg cm^{-2} SynII (Corning Inc., US) or 0.2–0.4 µg cm^{-2} rhBL (BioLamina, SE). For the study, uncoated discs acted as a negative control, while discs coated using 3 mL of GemCell™ Plus Xeno-Free Human Serum AB (GeminiBio, US) acted as positive controls. All coating procedures were performed in ultra-low attachment (ULA) 6-well plates (Corning Inc., US). Once coated, the discs were transferred to new 6-well ULA plates, where they were submerged in 3 mL of RI-supplemented E8F and inoculated with 5.0×10^4 cells cm^{-2}. The cells and discs were incubated for 24 h at 37 °C, 5 % CO_2, and 80 % relative humidity to permit cell attachment. Following the attachment phase, the discs were aseptically transferred to new 6-well ULA plates containing 3 mL of E8F. The hiPSCs were then incubated for a further 48 h before staining or harvest. Cell harvest was performed with TrypLE Select (Thermo

Fisher Scientific Inc., US) at 37 °C for 5–10 min. Once harvested, cell densities and EF were compared and statistical analyses performed using Prism 10 (Graphpad, US). Significance was determined using a one-way analysis of variance followed by Tukey's honest significance post-test. Differences were considered statistically significant if $p < 0.05$ (*).

Alongside the proteolytically treated cells and those subjected to label-free monitoring, cells were stained with fluorescent reagents to allow microscopic changes in cell confluence and distribution to be observed. Staining was performed by fixing the cells with 10 % neutral buffered formalin solution (Sigma-Aldrich, US), followed by permeabilization with a neutral buffered 0.3 % Triton X-100 solution (Sigma-Aldrich, US). Once permeabilized, the fixed cells were treated with a neutral buffered solution containing 5 μg mL^{-1} 4′,6-diamidino-2-phenylindole or DAPI (Roche, CH). Thereafter, the cells were stained with a 2 μg mL^{-1} neutral buffered propidium iodide [PI] (Miltenyi Biotec, DE). All steps were performed in 30 min intervals at 20–25°C. Once stained, the cells were gently washed with PBS (Sigma-Aldrich, US) and analyzed using the EVOS™ FL 2 Auto.

The results of the static studies confirmed that CAM application was necessary if hiPSC attachment to and growth on the PET-based discs was to be ensured under serum-free conditions (Figure S1A). Microscopic evaluation of the discs following fluorescent staining revealed a qualitatively similar confluence of ≈ 30 % between the SynII- (Figure S1B) and rhVTN-coated (Figure S1C) discs directly prior to harvest, while the rhBL- (Figure S1D) and serum-coated (Figure S1E) discs achieved almost complete confluence within the same time.

Subsequent cell harvest from the recombinant protein-based CAMs proved simple, while microscopic images taken of the discs following harvest confirmed significant cell presence on the SynII coated discs despite prolonged exposure to proteolytic treatment. Poor cell detachment from the SynII coated discs was attributed to the > 10-fold higher concentration in which the CAM was applied combined with the greater number of binding sites per unit mass (Suzuki et al. 1985; Martin et al. 2012).

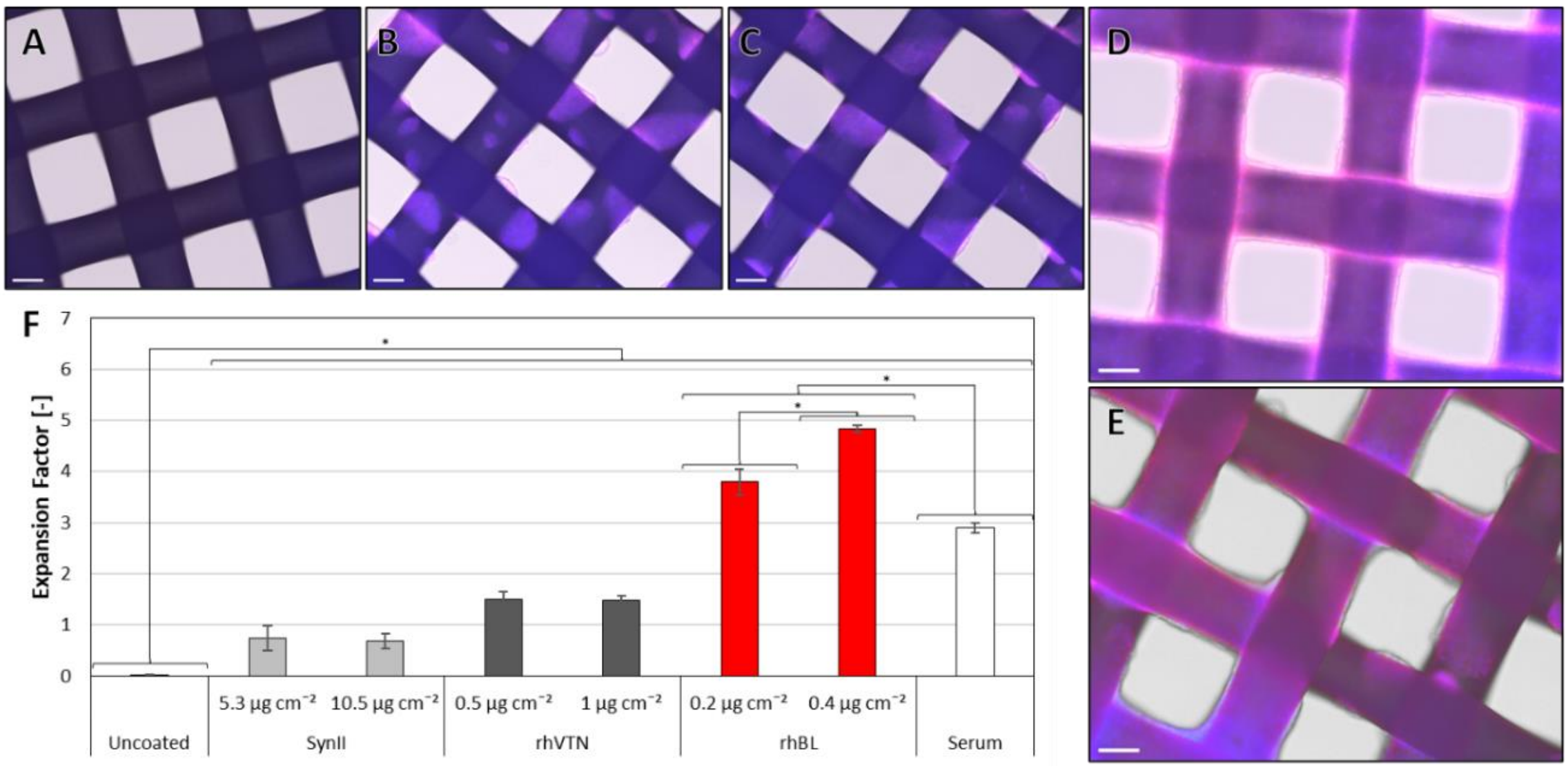

Figure S1: Evaluation of CAM-mediated hiPSC attachment and growth on PET-based discs in E8F prior to and following harvest. Qualitative evaluation of cell attachment, distribution, and growth to the (A) uncoated, (B) SynII-, (C) rhVTN-, (D) rhBL-, and (E) serum-coated discs was achieved through the application of fluorescent dyes, while (F) dose-dependent CAM potency was quantified through cell count following 3 d of cultivation. The scale bar in the lower left corner corresponds to 100 µm. * $p < 0.05$.

Remarkable discrepancies between CAMs based on their concentration during coating were also noted when evaluating the EF post-harvest (Figure S1F). More specifically, it could be demonstrated that the concentration of rhBL during coating correlated with subsequent cell attachment and growth, achieving EF of up to 5 and, thus, aligning with what has been reported in literature (Miyazaki et al. 2017) and improving on the serum-treated control both qualitatively (Figure S1D and E) and quantitatively by up to 70 % (Figure S1F). No such correlation could be observed for the rhVTN and SynII coatings, however. To determine the applicability of these findings to the dynamic cultivation of

hiPSCs, we selected the lowest concentration evaluated in each case for further testing in the AS1.
